# Common Disease: Are Causative Alleles Common or Rare?

**DOI:** 10.1371/journal.pbio.1001009

**Published:** 2011-01-18

**Authors:** Robert Shields

**Affiliations:** Public Library of Science, Cambridge, United Kingdom

It has been said that a week is a long time in politics. But in human disease gene mapping, 10 years can seem a very short time indeed. It once seemed so simple: find a family with a number of affected individuals and narrow down regions of the genome shared by affected individuals but not their unaffected siblings. This process (family linkage analysis) was lengthy but had notable success with some diseases, including hereditary breast cancers caused by the *BRCA1* and *BRCA2* genes. Yet, many diseases known to have a genetic component (because they tend to run in families or siblings show a high concordance) do not follow a simple Mendelian pattern of inheritance and cannot be dissected in this way. Instead, researchers tried an “association” approach, starting with a large number of unrelated individuals, to find gene variants, or alleles, that are more common in affected than in unaffected controls. For such a strategy to work, the diseases must be influenced by variants that are quite common in the populations.

Some readers might recall the heated debates about the common disease, common variant (CD-CV) hypothesis. Using arguments based on population genetics (such as the rate of creation and purging of deleterious alleles, the genetic bottleneck in the human population and subsequent population expansion) [1], the CD-CV hypothesis proposed that in common diseases with a genetic component, some predisposing alleles are relatively common and a combination of alleles or environmental effects was required before disease occurred, much like being dealt a bad hand from a common deck of cards. Under this hypothesis, disease-associated alleles might be found by using common gene variants, such as single nucleotide polymorphisms (SNPs), as a guide and comparing affected individuals with controls. Others cast doubt on this idea and suggested that common diseases are unlikely to be caused by common alleles and more likely to be caused by rarer ones; they too deployed arguments based on population genetics and suggested that association studies using common genetic variants might not be successful [2],[3]. As with all scientific debates, there seemed only one way out: collect the data and see. Well, 10 years and several millions of dollars later we have a lot of data, but are we any the wiser? Do we understand the allelic spectrum of disease any better than we did 10 years ago?

There have now been over 700 genome-wide association studies (GWAS) published linking many variants to over a hundred diseases [4]. Many of these results are robust in that they can be replicated in several populations, leaving little doubt that common variants can contribute to common diseases. The problem is that the effect of these variants on disease is often rather modest, so that people with the disease-predisposing alleles are only slightly more likely to get the disease than those without. Larger and larger studies reveal more disease genes, usually with smaller and smaller effect on overall disease risk. The “missing heritability” problem then arises because, even in aggregate, these loci typically fall somewhat short of explaining the entire genetic component of disease risk. So where are the genes accounting for major predisposition to disease? One possible explanation is that GWAS do not directly reveal the disease-causative DNA variant, but rather a common DNA variant (usually an SNP) that is close enough to be genetically linked to it (almost always inherited together) and common enough to be on the genotyping microarrays. This has spurred more effort (and more expense) to find rarer and rarer SNPs by sequencing more genomes and make even larger arrays in the hope that the new SNPs may be in even closer linkage with the causative allele. Alternatively, it's possible that the disease-predisposing variants are not SNPs at all, but other changes in the genome, such as a duplicated or deleted gene or region—a so-called copy number variant (CNV)—or a result of epigenetic marks in the chromatin; neither of these would show up using the current generation of microarrays that look just at SNPs.

A paper published recently in *PLoS Biology* [5] from the lab of David Goldstein put the cat amongst the CD-CV pigeons by suggesting that rather than common diseases being caused by common alleles, maybe rare alleles each with a large effect on disease might be creating “synthetic associations” in the GWAS signal by occurring, by chance, more often with one common allele than another. The paper used statistical reasoning to suggest that such synthetic associations are possible—but are they likely? Given how much time and money has been invested in surveying SNPs and attempting to match them up to diseases, the relative importance of such synthetic associations would have important implications on the direction of future research. The paper got a lot of publicity, even making the *New York Times* [6].

Now, some might say that no one likes the implication that they have been barking up the wrong scientific tree, still less perhaps that such a critique garnered a lot of publicity. But the issue is best settled by discussion—and data—which is why in this issue of *PLoS Biology* we publish two critiques of the original article [7],[8] together with a response from the original authors [9]. The critiques argue that although rare variants could in theory create synthetic associations, this is not a likely explanation for the missing heritability. Perhaps with further advances in ever cheaper sequencing technologies and the ability to sequence whole genomes from affected individuals we will, before the next 10 years are up, finally have a better understanding of the missing pieces of the genetic causes of common disease.

## Abbreviations

CD-CV: common disease, common variant
CNV: copy number variant
GWAS: genome-wide association studies
SNP: single nucleotide polymorphism
